# Ascorbate Oxidase Induces Systemic Resistance in Sugar Beet Against Cyst Nematode *Heterodera schachtii*

**DOI:** 10.3389/fpls.2020.591715

**Published:** 2020-10-22

**Authors:** Richard R. Singh, Neriza Nobleza, Kristof Demeestere, Tina Kyndt

**Affiliations:** ^1^Department of Biotechnology, Faculty of Bioscience Engineering, Ghent University, Ghent, Belgium; ^2^Research Group Environmental Organic Chemistry and Technology (EnVOC), Department of Green Chemistry and Technology, Ghent University, Ghent, Belgium

**Keywords:** ascorbate oxidation, phenylpropanoid, hydrogen peroxide, systemic defense response, cyst nematodes (*Heterodera* spp.)

## Abstract

Ascorbate oxidase (AO) is an enzyme involved in catalyzing the oxidation of apoplastic ascorbic acid (AA) to dehydroascorbic acid (DHA). In this research, the potential of AO spraying to induce systemic resistance was demonstrated in the interaction between sugar beet root and cyst nematode *Heterodera schachtii* and the mechanism was elucidated. Plant bioassays showed that roots of AO-sprayed plants were infested by a significantly lower number of females and cysts when compared with mock-sprayed control plants. Hormone measurements showed an elevated level of jasmonic acid (JA) salicylic acid (SA) and ethylene (ET) in the roots of AO-sprayed plants, with a dynamic temporal pattern of activation. Experiments with chemical inhibitors showed that AO-induced systemic resistance is partially dependent on the JA, ET and SA pathways. Biochemical analyses revealed a primed accumulation of hydrogen peroxide (H_2_O_2_), and phenylalanine ammonia lyase (PAL) activity in the roots of AO-sprayed plants upon infection by cyst nematodes. In conclusion, our data shows that AO works as an effective systemic defense priming agent in sugar beet against cyst nematode infection, through activation of multiple basal plant defense pathways.

## Introduction

Sugar beet (*Beta vulgaris* L.) is the primary source of sugar in the temperate zone, and it accounts for 20% of the world’s total sugar production. The EU is the world’s leading sugar beet producer with 16.84 million tons of sugar production in 2019 (FAOSTAT, 2019). While fungal diseases such as Cercospora leaf spot, rust, powdery mildew and Ramularia leaf spot are the most common aboveground diseases negatively affecting sugar beet yield (Heick et al., 2020; Rangel et al., 2020), belowground plant-parasitic-nematodes (PPN) equally contribute to damage in sugar beet production (Cooke, 1987, 1993). Generally, crop losses caused by PPN have been estimated at $US80 billion per year globally (Nicol et al., 2011). *Heterodera schachtii* [beet cyst nematode (BCN)] is responsible for 90% of all nematode-related sugar beet infestations worldwide (Robb et al., 1992; Agrios, 2005). In addition to direct damage caused by BCN, penetration of nematodes in the roots cause an entry point for infection by other pathogens such as *Rhizoctonia*, viruses, and *Cercospora* spp. (Back et al., 2002; Agrios, 2005). BCN are sedentary endoparasites feeding within the roots of their host (Moens et al., 2018). The root exudates provides a cue for infective second-stage juveniles (J2) to hatch which then invade the roots in the elongation zone behind the root tips (Wyss and Grundler, 1992; Moens et al., 2018). Upon invasion, the J2 migrate into the cortex after which they initiate a feeding structure (syncytium) in the root vascular tissue (Sobczak and Golinowski, 2009). The syncytium is formed by the breakdown of plant cell walls and subsequent fusion of adjacent protoplasts, resulting in a large multinucleate cell, and provides nutrients for the developing nematodes (Grundler et al., 1998; Kyndt et al., 2013; Anjam et al., 2020). After fertilization, around 200–300 eggs are laid, and once fully developed, the female dies, and its cuticle hardens to form a brown cyst (Khan et al., 2016). Cysts have the ability to survive in the soil for many years in the absence of a suitable host plant, making it difficult to control (Perry et al., 2018).

Protection of sugar beet against BCN is currently based on agronomic measures such as growing tolerant varieties or application of nematicides. Crop tolerance is a useful and necessary adjunct to resistance (Trudgill, 1991) as tolerance reduces the negative effects of pathogens, simultaneously improving plant growth after infections has occurred (Koch et al., 2016). For example, Eberlein et al. (2020) showed control of *H. schachtii* by growing the tolerant Pauletta cultivar. As another possible alternative for nematicides, so-called “induced resistance (IR)” of the plant provides a more sustainable solution, potentially addressing the EU regulations to protect our environment. The most efficient way of IR works through defense priming. Defense priming has two key phases whereby (1) a priming stimulus (also known as priming agent or elicitor) slightly awakens or activates defense responses when applied to plants, and (2) when challenged with a triggering stimulus (a stress factor), defense responses work in much faster and stronger manner than in naïve plants (Martinez-Medina et al., 2016). Priming hence puts a plant in a state of increased alertness with no or minimal gene induction, and no energy and yield loss (Conrath et al., 2015). Plant defense priming has a low fitness cost, activates robust defense responses with broad spectrum activity, and has a low ecological cost (Martinez-Medina et al., 2016). One such defense elicitor is β-aminobutyric acid (BABA), a non-protein amino acid that can activate plant defense to defeat a variety of subsequent stress factors (Conrath et al., 2006, 2015). For example, BABA induces resistance in potato against *Phytophthora infestans* (Bengtsson et al., 2014). Reduced numbers of cyst nematodes *H. avenae* and *H. latipons* were observed on wheat and barley upon foliar sprays with BABA (Oka and Cohen, 2001). However, a major drawback of this defense elicitor is that there have been records of phytotoxic effects on some crop plants (van Hulten et al., 2006; Koen et al., 2014; Luna et al., 2016). Recently, a novel registered defense elicitor—combining chitosan oligomers (COS) and pectin-derived oligogalacturonides (OGA), called COS-OGA—was shown to control mildew attack in grapes, cucumbers, tomatoes (van Aubel et al., 2014; Van Aubel et al., 2016), and *P. infestans* in potato (Van Aubel et al., 2018) and very recently a COS fluorinated derivative chitosan-thiadiazole-trifluorobutene (COSSZFB) was shown to control root knot nematode (RKN) *Meloidogyne incognita* in cucumber seedlings (Fan et al., 2020). In our previous research, this mixture has also been demonstrated to control RKN *M. graminicola* in rice, through systemic activation of the plant phenylpropanoid pathway (Singh et al., 2019). Additionally, silicon (Zhan et al., 2018) and thiamine (Huang et al., 2016) were shown to activate rice defense against *M. graminicola.* In sugar beet, thiamine (Vitamin B6) has been shown effective to activate plant defense against the fungal pathogen *Rhizoctonia solani* (Taheri and Tarighi, 2011), via jasmonate-mediated priming of the phenylpropanoid pathway. However, the use of these or other IR compounds to control BCN in sugar beets has not yet been evaluated.

Naturally, plants express a wide array of defense responses against numerous groups of abiotic and biotic stressors. Plant hormones such as salicylic acid (SA), jasmonic acid (JA) and ethylene (ET) are the central hubs of plant basal immunity (Spoel and Dong, 2008; De Vleesschauwer et al., 2013). ET is known to play a role against RKN infection through activation of JA (Nahar et al., 2011; Mantelin et al., 2017). The JA and SA pathways are known to play a pivotal role for example in Arabidopsis against infection of BCN (Kammerhofer et al., 2015), and in rice defense against *M. graminicola* infection (Nahar et al., 2011; Kyndt et al., 2017). Nematodes have been shown to suppress hormones during a compatible interaction with their host, to attain plant susceptibility. For example, RKNs suppress the SA and ET pathways in infected rice plants (Kyndt et al., 2012), and recent transcriptome data from our lab (Ghaemi et al., in press) provided evidence for ET suppression by BCN in sugar beet. While some hormonal pathways are suppressed by nematodes, others are important for feeding site and nematode development—for example auxin and cytokinin are required for feeding site formation (Gheysen and Mitchum, 2011; Kyndt et al., 2016; Gheysen and Mitchum, 2019). SA biosynthesis in plants follows two pathways namely isochorismate (IC) or the phenylalanine ammonia-lyase (PAL) pathways. The first step of the phenylpropanoid metabolism is the conversion of L-phenylalanine into trans-cinnamate, catalyzed by PAL (MacDonald and D’Cunha, 2007). Increases in PAL activity are often detected in the early response of plants upon pathogen attack (Giberti et al., 2012). In a recent study, JA was shown to protect tomato from RKN infection via a systemic signaling pathway, whereby root infection by nematodes causes signal transmission to the shoots, to induce biosynthesis of JA which is then transported to the root to induce defense against RKN *M. incognita* (Wang et al., 2019). This indicates that communication between tissues (shoot to root and vice versa) are vital for systemic defense signaling.

Reactive oxygen species (ROS) accumulate upon plant encounters with pathogens in a process called the oxidative burst. The most stable ROS, H_2_O_2_, also has the capacity to act as a signaling molecule (Smirnoff and Arnaud, 2019), playing positive roles in the plant cell (Foyer and Noctor, 2005; Foyer, 2018). At high concentrations this molecule is cytotoxic, and hence antioxidants are needed to protect cells from oxidative damage. Ascorbic acid (AA) also known as Vitamin C, is such a non-enzymatic antioxidant contributing to ROS-scavenging (Foyer and Noctor, 2011). This molecule is known to play a role in plant defense against many pathogens (Mittler et al., 2004), for example in resistance against several PPN. Arrigoni et al. (1979) showed that the application of AA increased tomato resistance against *M. incognita*. Likewise, Zacheo et al. (1981) showed an increased level of AA in resistant pea plants when challenged by the cyst nematode *H. goettingiana.* In our recent transcriptome analysis of sugar beet upon infection with BCN, we similarly observed strong induction of AA-related genes in resistant plants (Ghaemi et al., in press).

AA is a water-soluble antioxidant that is universally distributed in higher plants, where it acts as a cofactor of many enzymes, and is known to be present in all compartments of plant cells in mM concentrations (Anjum et al., 2014; Foyer et al., 2020). In the apoplast, AA becomes oxidized during the oxidative burst. The reduction/oxidation (redox) state of the apoplastic AA pool is known to be regulated by ascorbate oxidase (AO) (Pignocchi et al., 2003; Foyer et al., 2020), a glycoprotein and a member of the blue copper oxidase family. In the apoplast, reduced AA concentration is lower while the oxidized form of AA, dehydroascorbate (DHA), is more predominant than in the symplast (Smirnoff and Arnaud, 2019). The breakdown of DHA results in the formation of derivatives, such as oxalic, threonic acid and 2,3-L-diketogulonate in the apoplast (Dewhirst and Fry, 2018), which leads to generation of H_2_O_2_ that could play a role in defense signaling (Smirnoff, 2018). AO was shown to play a role in cell growth (De Tullio et al., 2013) and work on transgenic AO overexpression lines demonstrated that AO plays a role in stress tolerance (Yamamoto et al., 2005; Pignocchi et al., 2006; Garchery et al., 2013; Karpinska et al., 2018). Recently, we elucidated the role of AA oxidation in rice defense responses against RKN, and we discovered that foliar AO application can activate systemic plant defense in the rice roots without any negative effects on plant growth (Singh et al., 2020). The aim of this research was to investigate the potential of AO to also enhance defense responses in the evolutionary diverged dicot plants and against cyst nematodes, which have a rather different infection strategy than RKN. While RKN migrate in-between the cells when they enter the root tissue, CN move intracellularly toward the vascular tissue, causing more damage and hence activating plant defense responses. Next to that, feeding site formation by RKN involves giant cell formation, while CN induce a syncytium through cell wall dissolution (Kyndt et al., 2013). As dicot plant, we worked with the agronomically important sugar beet crop, which production is threatened by BCN infestation.

First, we investigated the susceptibility of AO-sprayed sugar beet plants to BCN. We quantified the alterations in AA and hormone concentrations in sugar beet roots at different time points upon foliar application of AO. Further, we evaluated the functional role of plant defense hormones SA, JA and ET in AO-induced defense, by using hormone inhibitors and gene expression analysis. Finally, we investigated changes in H_2_O_2_ and PAL activity during the migratory and early sedentary stage of nematode infection in AO-treated vs. naïve plants.

## Materials and Methods

### Plant Material and Growth Conditions

Sugar beet cv. Amarok seeds were germinated in moist potting soil for 4–5 days at 25°C. For short-term infection assays (to determine the number of penetrating juveniles) and for biochemical assays, seedlings were transplanted to PVC tubes containing a mixture of fine sand and synthetic absorbent polymer (SAP) substrate, as a growth medium (Reversat et al., 1999). For long-term infection assays (to determine the number of females and cysts) the seedlings were transplanted to polyvinyl-chloride (PVC) tubes containing fine soil particles. All experiments were conducted in a plant growth room at 25°C, with a 16:8 hours (h) light-regime. The plants were fertilized by supplying Hoagland solution (Hoagland, 1937) as a source of nutrients three times a week.

### Nematode Inoculation and Infection Assessment

Beet cyst nematode (BCN), *Heterodera schachtii* Schmidt (cysts originally obtained from Razieh Ghaemi, Iran) was cultured on sugar beet (cv. Amarok) plants. Cysts were obtained from stock cultures, by washing the infected soil, letting the cysts to float in water and subsequent hand-picking with a small paint brush. Full cysts with eggs were selected and allowed to dry before setting up for hatching. For second-stage juveniles (J2) to hatch, cysts were placed in 3 mM of ZnCl_3_ and transferred to an incubator/shaker at a temperature of 25.5°C at 70 rpm for 4 days (Ghaemi et al., in press). J2 were re-suspended in water and counted under a stereomicroscope. Around 300 J2 nematodes were inoculated per plant 3 weeks after transplanting. One day before and after inoculation, plants were not supplied with any nutrients or water.

To count the number of J2/J3, a subset of plants (*n* = 8) were collected at 4-days post inoculation (dpi). To visualize the J2/3 nematodes inside the roots, the nematode-infected roots were washed and boiled for 3 min in 0.8% acetic acid and 0.013% acid fuchsin solution, followed by destaining in acidified glycerol. To count the number of females, a second subset of plants (*n* = 8) were collected at 24 dpi; and to count the number of cysts, a third subset of plants (*n* = 8) were harvested at 6 weeks post inoculation (wpi). Cysts, collected as described above, were categorized either as empty (no eggs) or full cysts (with eggs) using a stereomicroscope (Leica microsystem). J2/J3 were counted under a stereomicroscope, while females and cysts were counted visually. For all infection experiments, plant shoot height (SH), root length (RL), fresh shoot weight (FSW) and fresh root weight (FRW) were measured, and the plants were visually observed to rule out any negative effects of spraying with AO on plant growth. All experiments were independently repeated at least three times, except for the penetration count at 4 dpi, which was only done twice, each time with eight plants per treatment.

### Spraying With Chemicals and Hormone Biosynthesis Inhibitors

The following chemicals and hormone biosynthesis inhibitors with respective concentrations were used: Ascorbate oxidase (AO; Sigma-Aldrich, Merck, Darmstadt, Germany) at 5, 10, and 20 U/mL (Singh et al., 2020), aminooxyacetic acid, a potent inhibitor of ET biosynthesis (AOA; Sigma-Aldrich, Merck, Darmstadt, Germany) at 30 mM (Nahar et al., 2011; Kammerhofer et al., 2015), L-2-aminooxy 3-phenylpropanoic acid, a potential inhibitor of PAL activity (PAL-Inh; Wako-chemicals, Osaka, Japan) at 10 mM (Kammerhofer et al., 2015) and lipoxygenase-inhibitor diethyldithiocarbamic acid (DIECA; Sigma-Aldrich, Darmstadt, Germany) at 100 μM (Nahar et al., 2011). All chemicals were dissolved in water except for PAL-Inh which was dissolved in 1 mL of EtOH before diluting further in water. All chemicals were foliar sprayed and a surfactant, 0.02% (v/v) of Tween20 was added to all spraying solutions to allow efficient uptake (Nahar et al., 2011). Control plants were mock-sprayed with distilled water containing 0.02% (v/v) of Tween20. In each experiment, 3-weeks-old plants were sprayed until run-off with 6.25 mL of solution. This was done at 24 h prior to nematode inoculation in case where infected plants were used. All chemicals have been optimized in previous publications (see above) with chemical concentrations tested for bio-efficacy and lack of phytotoxicity.

### Hormone Measurements

Whole root material was sampled from four biological replicates, each consisting of a pool of four individual plants. Root material was collected 12 and 24 h after foliar applications.

#### Measurement of IAA, ABA, SA, and JA

A cold extraction of 100 mg of homogenized root material was performed using the modified Bieleski solvent, followed by filtration and evaporation (Haeck et al., 2018). Chromatographic separation of the extracted phytohormones was achieved on an ultra-high-performance liquid chromatography (U-HPLC) system (Thermo Fisher Scientific, San Jose, CA, United States), equipped with a Nucleodur C18 column (5092.1 mm, 1.8 lm) and using a mobile phase gradient consisting of acidified methanol and water. High-resolution mass spectrometric analysis (HRMS) was carried out in a full-scan mode using a Q-Exactive^TM^ Orbitrap mass spectrometer (Thermo Fisher Scientific), applying heated electrospray ionization in polarity switching mode and operating at a mass resolving power of 70 000 full width at half-maximum (Haeck et al., 2018).

#### Measurement of ET

ET content was determined according to López-Gálvez et al. (2015). A gas chromatograph with flame ionization detector was used (GC-FID) (Finnigan Trace GC Ultra, Thermo Fisher Scientific, Waltham, United States) equipped with the columns15 m/0.53 mm/RT-MSieve 5A (Restek, Bellefonte, United States), 30 m/RTU-Piot (Restek), and 25 m/0.53 mm/CP-PoraBOND Q Fused Silica (Varian, PaloAlto, United States). After calibration of the equipment, samples (∼1 mL) of headspace from weighed chopped material were injected into the column using a gas syringe. The GC oven was set at a constant temperature of 35°C, the carrier gas was helium and the FID used pressurized hydrogen and air. The software Thermo Finnigan Chrom Card32-bit was used to interpret the results.

### Evaluation of Priming of Defense Responses

To evaluate a possible primed defense response, we set up a multifactorial experiment including four groups: (1) naïve plants uninfected (2) AO-sprayed uninfected, (3) naïve plants infected, and (4) AO-sprayed infected plants. Three-weeks-old plants were sprayed with AO (20 U/mL), naïve plants were mock-sprayed. Plants were either uninfected or infected with nematodes 24 h after spraying with AO or mock-sprayed. Roots and shoots of all plants were collected at 4 and 24 dpi. A total of four biological replicates per treatment were used, and each biological replicate contained a pool of roots or shoots from 4 to 5 plants. The samples were immediately frozen in liquid nitrogen (N_2_) and ground to fine powder using a mortar and pestle.

#### H_2_O_2_ Measurement

Hydrogen peroxide level was determined according to Velikova et al. (2000). For each sample, about 100 mg of powder was dissolved in 800 μL of 0.1% (w/v) trichloroacetic acid. The homogenate was centrifuged at 14,000 rpm, at 4°C for 15 min. 60 μL of the supernatant was added to 60 μL of 10 mM potassium phosphate buffer (pH 7.0) and 60 μL of 1 M KI. The absorbance of the supernatant was read at λ_*max*_ = 390 nm. Hydrogen peroxide content was calculated based on a standard curve made by measuring known hydrogen peroxide concentrations in the same assay.

#### PAL Activity Measurement

PAL activity was measured according to Camacho-Cristóbal et al. (2002). Hundred mg of each sample was dissolved in 800 μL of 50 mM sodium phosphate as assay buffer containing 2% (w/v) poly vinylpolypyrrolidone (PVPP), 2 mM ethylenediaminetetraacetic acid (EDTA), 18 mM-mercaptoethanol and 0.1% (v/v) Triton X-100. The homogenate was centrifuged at 8,000 rpm, at 4°C for 10 min. In different 2 mL tubes, 135 μL of reaction buffer, 50 μL of 5 mM of L-phenlyalanine, and 20 μL of supernatant were mixed. Absorbance measurement was done using a spectrophotometer at 290 nm. The sample was then incubated in the water bath for 30 min at 40°C, after which 10 μL of hydrochloric acid was added and mixed for 10 min. PAL was assayed by measuring the formation of trans-cinnamic acid at 290 nm. One unit (U) of PAL-activity was defined as the amount of the enzyme that produced 1 nmol trans-cinnamic acid per hour. Negative control reactions had no L-phenylalanine as substrate.

### Ascorbic Acid Measurement

Plant root and shoot samples were collected from a pool of 4–5 plants per treatment and crushed in liquid nitrogen. In each assay, 100 mg of crushed material was used per sample. For each measurement, at least 5 biological replicates were analyzed per treatment. Reduced and oxidized AA were measured according to Ueda et al. (2013) and Wu et al. (2017) with slight modifications. Samples were extracted using 6% (w/v) metaphosphoric acid containing 1 mM EDTA. Reduced AA concentration was measured in presence of 100 mM potassium phosphate buffer (PPB) (pH 7.0) and 0.1 units AO. Oxidized AA was measured with sample containing PPB (pH 7.8) and dithiothreitol. Absorbance was monitored in a microplate reader (TECAN- Spectrophotometer) at 265 nm in the UV-transparent 96-well microplates for 8 mins (ε = 14.3 mM^–1^ cm^–1^).

### Quantitative Reverse Transcriptase PCR (qRT-PCR)

For qRT-PCR, 3 weeks old plants were sprayed with AO and water. Root and shoot tissues were collected at 24 h after spraying for RNA-extraction. A total of four biological replicates and two technical replicates were used. RNA was extracted using Qiagen RNeasy Plant Mini Kit (Hilden, Germany). The RNA concentration and purity were evaluated using the NanoDrop 2000 spectrophotometer. A total of 1 μg of each RNA sample was treated with 1 U of DNaseI enzyme (Thermo Fisher Scientific). The cDNA synthesis was performed using 200 U of Tetro Reverse Transcriptase enzyme and Oligo (dT)_18_ primer (Tetro cDNA Synthesis kit, Bioline, Germany) according to the manufacturer’s protocol. Quantitative PCR was performed using SensiMix^TM^ SYBR NO-ROX (Bioline, Germany) on a CFX connect real-time PCR machine (Biorad, United States) as following: 10 min of initial denaturation at 95°C and 40 amplification cycles (25 s at 95°C, 25 s at 58°C, and 20 s at 72°C). After the last step, specificity was tested using a melting curve by gradually increasing the temperature to 95°C. Data were analyzed using Rest 2009 (Pfaffl et al., 2002). The investigated genes included sugar beet orthologs of Arabidopsis markers for the salicylic acid (SA) pathway (*PATHOGENESIS-RELATED1; PR1*), and genes associated with the ET (*RELATED TO AP2–3; RAP2–*3), JA (*LIPOXYGENASE2; LOX2*) pathways, as developed by Schmidt et al. (2020). Expression levels were normalized using two reference genes, *glyceraldehyde-3-phosphate dehydrogenase (GAPDH*) and *Isocitrate dehydrogenase (ICDH)*. All primers used in this study are listed in Schmidt et al. (2020).

### Nematicidal Assay

To evaluate if AO has any direct effect on the nematodes, a nematicidal assay was performed. One hundred J2’s was placed in eight counting wells (*n* = 8) with 20 U/mL AO or with water (control). The plates were kept in the dark at room temperature. The number of dead (inactive) and alive (active and moving) nematodes (J2) were counted at a 1-day interval for 4 days. Results were presented as total number of living nematodes out of the total number of nematodes (%).

### Data Collection and Statistical Analysis

All statistical analyses were performed using the software SPSS (version 21). The normality of data was checked by the Kolmogorov-Smirnov test of composite normality (α = 0.05). Homogeneity of variance was checked by the Levene test (α = 0.05). Analysis of variance (one-way ANOVA) and multiple comparisons of differences between treatments were then performed by Duncan’s multiple range test (α = 0.05). Comparisons between two means were conducted using a two-tailed Student’s *t*-test.

## Results

### Foliar Application of AO on Sugar Beet Reduces *Heterodera schachtii* Infection in the Roots

Foliar application of AO (20 U/mL) has previously been found to reduce the susceptibility of rice plants to *M. graminicola* infection (Singh et al., 2020), and the transcriptional response provided evidence for a primed ET/JA-dependent defense response. Here, we investigated the potential of AO application on dicot sugar beet to protect this crop plant from BCN infection. In the first preliminary experiment, the dose response effects of AO on sugar beet against *H. schachtii* infection were evaluated. In comparison with control, plants sprayed with 10 U/mL, and 20 U/mL of AO had significantly reduced number of cysts (38% and 60% reduction respectively) (Supplementary Figure S1A). No significant changes in plant growth were observed in the treated plants when compared with control (Supplementary Figures S1B–E). At a concentration of 20 U/mL, a significant increase in FRW was observed in AO-treated plants when compared with control plants (Supplementary Figure S1E). Thus, in further experiments we choose to work with a concentration of 20 U/mL, because of the strongest negative effect on BCN infection and positive effect on root growth.

**FIGURE 1:**
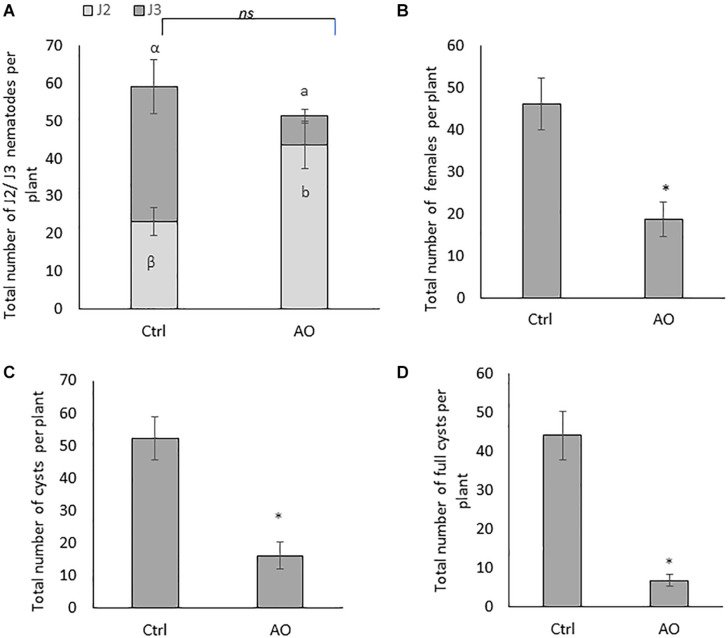
Effect of foliar spraying of AO on *Heterodera schachtii* infection in sugar beet roots. Three-weeks-old plants were sprayed with 20 U/mL AO, while control plants were mock sprayed. One day later, plants were inoculated with 300 second-stage juveniles (J2). Plant responses were analyzed at different time points for different stages of the nematode infection cycle. Total number of **(A)** penetrated juveniles at 4 dpi (J2 and J3), **(B)** females at 21 dpi, **(C)** cysts, and **(D)** full cysts at 6 wpi. Data for **(A)** was analyzed by one-way ANOVA and Duncan’s multiple range test (α = 0.05). Data for **(B–D)** were analyzed using a two-tailed Student’s *t*-test. Different letters and asterisks indicate statistically different means. Bars represent means ± SD of a total of 8 individual plants (*n* = 8).

In the second experiment, plant susceptibility was evaluated by counting the number of J2 and J3 at 4 dpi, the total number of females at 24 dpi, and the total number of cysts (empty or full) at 6 wpi. Foliar application of 20 U/mL AO did not significantly affect the total number of penetrated juveniles (J2 + J3) when compared with mock-sprayed plants (Ctrl) (Figure 1A). However, a significantly higher number of J2 in AO-sprayed plants were observed when compared with mock-sprayed plants, and a concomitant decrease of J3 in AO vs. mock- sprayed plants (Figure 1A). This suggests that although an equal number of nematodes could penetrate the roots, their development is delayed in AO-sprayed plants compared with control plants already at this early time point.

At later time points, AO-sprayed sugar beet plants contain a significantly reduced number of females (60% reduction at 24 dpi) (Figure 1B) and a significantly reduced number of cysts (70% reduction) at 6 wpi (Figure 1C). The total number of cysts is not a conclusive indicator for successful control of BCN. Estimating the viable cysts, filled with eggs, is crucial for evaluating effective control measures (Christoforou et al., 2014). Therefore, we counted the number of full cysts, in AO-sprayed vs. mock-sprayed plants. Our result shows a significantly reduced number of full cysts (86% reduction) in AO-sprayed plants when compared with control plants (Figure 1D), confirming that AO spraying compromises nematode reproduction on the sugar beet host.

To further investigate whether application of AO has any effects on plant growth, we evaluated RL, SH, FSW and FRW in plants at 4 dpi, 24 dpi, and 6 wpi. No differences in the phenotype between the mock-sprayed and AO-sprayed plants were observed at 4 dpi (Supplementary Figure S2). Also, no negative effects of AO were observed at 24 dpi (Supplementary Figure S3) and 6 wpi (Supplementary Figure S4). Interestingly, a significant increase in SH (Supplementary Figures S3A,E) and root weight (Supplementary Figures S3D,E) was observed in AO-sprayed plants at 24 dpi and a significant increase in FRW (Supplementary Figures S4D,E) was observed in AO-sprayed plants at 6 wpi. To determine if AO has any direct effect on nematode mortality, we set up a nematicidal assay whereby J2 were incubated in AO or water, and their viability was counted at a 1-day interval for 4 days. We did not observe any significant differences in J2 viability between the AO incubated and water incubated groups, consistently up to 4 days of exposure (Supplementary Figure S5). Taken together, these data indicate that AO activates systemic induced resistance against BCN in sugar beet.

### AO Shoot Spraying Causes Accumulation of DHA in Roots

To investigate if AO application causes changes in the levels of reduced and oxidized AA (DHA), we measured their levels at 24 h after AO foliar application. Basal level of total ascorbic acid (mostly reduced form of AA) was higher in shoots than in roots (Supplementary Figure S6). Significantly higher levels of DHA were observed in roots of AO-sprayed plants (Supplementary Figure S6) suggesting that AO has a systemic signaling effect, causing accumulation of DHA in roots. The observation that AA levels are depleted upon AO application in the shoots suggests systemic transport from shoot to root.

### AO-Induced Defense in Sugar Beet Is Partially Mediated by Defense Hormone Pathways

**FIGURE 2:**
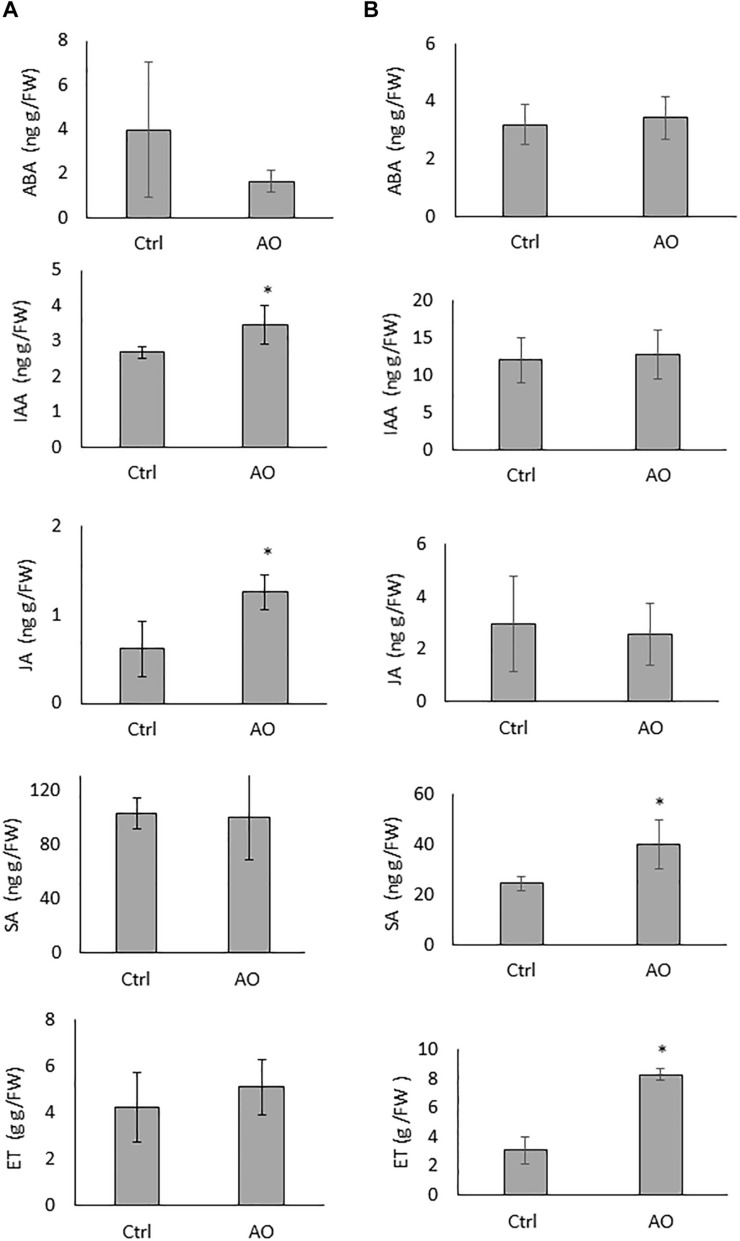
Effect of foliar AO spraying on phytohormone accumulation in sugar beet roots. Hormone ABA, IAA, JA, SA, and ET content in the roots of plants sprayed with (AO, 20 U/mL) or mock sprayed (Ctrl) at **(A)** 12 h and **(B)** 24 h after treatment. Data was analyzed using a two-tailed Student’s *t*-test (α = 0.05). Asterisks indicate statistically different means. Values presented are the average ± SD of five biological replicates each consisting of at least four individual plants. ABA, abscisic acid; IAA, indole-3-acetic acid; JA, jasmonic acid; SA, salicylic acid, ET, ethylene.

To evaluate if foliar AO application on sugar beet plants induces systemic changes in phytohormone levels, roots of AO-sprayed, and mock-sprayed plants were collected 12 and 24 h after spraying. Our results show that the concentrations of IAA and JA were significantly elevated at 12 h after spraying, while the concentrations of ABA, SA and ET were not changed in the roots of AO-sprayed plants at this time point (Figure 2A). At 24 h after spraying, the concentration of SA and ET were significantly elevated (∼2 and ∼4 times higher), while the concentrations of ABA, IAA and JA were not changed in the roots of AO-sprayed plants in comparison with mock-sprayed control plants (Figure 2B).

**FIGURE 3:**
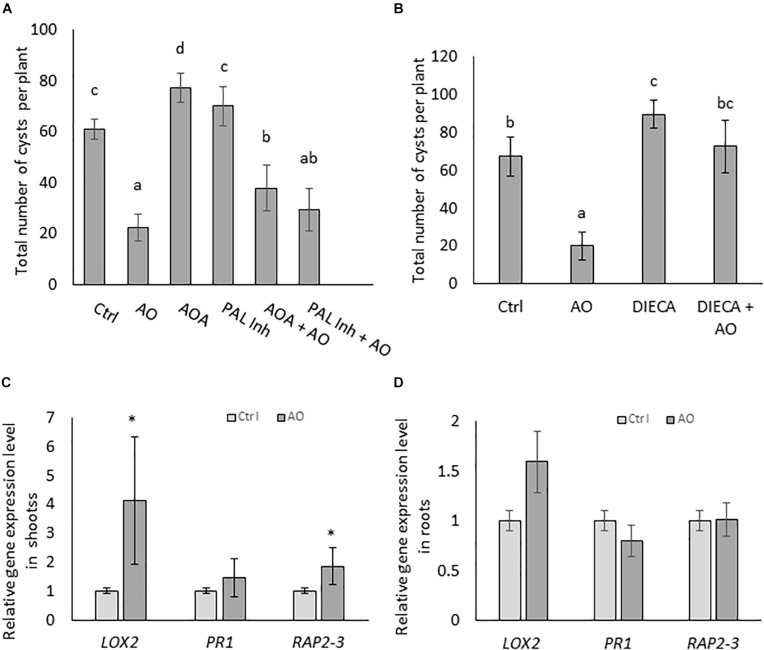
AO-induced defense against cyst nematodes is partially dependent on SA ET, and JA biosynthesis in sugar beet. Three-weeks-old sugar beet plants were sprayed with AO and chemical inhibitors. Plants were sprayed with **(A)** AO, AOA, AOPP (PAL-Inh) either alone or in combination and **(B)** AO, DIECA, and DIECA combined with AO. Water with Tween20 was sprayed as negative control (Ctrl). Plant roots were inoculated with 300 nematodes, 24 h after spraying with respective chemicals. The total number of cysts were counted at 6 wpi. Bars represent means ± SD of the number of cysts of eight plants. For **(A,B)** different letters indicate statistically significant differences (Duncan’s multiple range test with α = 0.05). Gene expression analysis with qRT-PCR on **(C)** shoots and **(D)** roots of AO treated sugar beet plants. The relative expression levels of JA-biosynthesis gene *LOX2*, SA-response gene *PR1*, and ET-responsive transcription factor *RAP2-3* were analyzed at 24 h after foliar treatment. Values presented are means ± SE of four biological replicates (each a pool of five individual plants) per treatment. Gene expression levels were normalized using two internal reference genes, *GAPDH* and *ICDH*. Data are shown as relative transcript levels in comparison with the control plants (expression level set at 1). In c and d asterisks indicate significant differential expression between AO-treated and control plants (REST-analysis; α = 0.05). AO, Ascorbate oxidase; AOA, aminooxyacetic acid; PAL-Inh, AOPP, L-2-aminooxy 3-phenylpropanoic acid.

To investigate if AO-induced systemic defense is dependent on increases in the three typical plant defense hormones, SA, JA and ET, we set up infection experiments with inhibitors of these pathways. Plants were foliar sprayed with hormone biosynthesis inhibitors separately and in combination with AO. In a first experiment, we inhibited SA and ET, because they were accumulating in roots upon AO spraying at 24 h (Figure 2B). AOA, an ET biosynthesis inhibitor and PAL-Inh, an inhibitor for phenylalanine ammonia lyase were used. To investigate infection severity, the number of cysts was counted at 6 wpi. Our results show a reduced number (64% reduction) of cysts in AO-sprayed plants (Figure 3A) which confirms our earlier data see (Figure 1C and Supplementary Figure S1A). A significant increase in the number of cysts was observed in plants sprayed with AOA when compared with the control plants, showing that ethylene plays a role in the basal defense of sugar beet against BCN. When plants were sprayed with PAL-Inh, there were no significant changes in the number of cysts compared with control plants. However, when AO was combined with AOA or with PAL-Inh, a lower but still significant decrease in number of cysts was observed when compared with plants sprayed with AOA or PAL-Inh alone (Figure 3A), showing that AO is still partially active. Based on the observation of JA accumulation at 12 h after AO spraying, we decided to execute a second experiment, to evaluate the role of JA biosynthesis in AO-induced defense against BCN. Upon foliar application of JA biosynthesis inhibitor DIECA a significant increase in number of cysts was observed, showing that LOX-mediated JA production plays a role in sugar beet defense against BCN (Figure 3B). Plants sprayed with DIECA + AO showed no significant difference in cyst numbers when compared with control plants. This suggests that AO-induced defense is dependent on LOX-mediated JA production.

To investigate this further, gene expression levels of sugar beet orthologs of three well-known Arabidopsis SA, JA and ET marker genes (developed by Schmidt et al., 2020) were monitored by qRT-PCR on AO-treated and control plants. The results revealed significantly higher expression levels of *LOX2* (JA biosynthesis) and *RAP2-3* (ET responsive transcription factor) in the shoot tissues of AO-treated plants at 24 h after spraying (Figure 3C). In the root tissue, a slight induction of the *LOX2* gene was observed in the roots of AO sprayed plants (Figure 3D). This observation of a predominant *LOX2* gene induction in shoots correlates well with the recent study of Wang et al. (2019) who demonstrated that the JA needed for tomato defense against root-knot nematodes is produced in aboveground tissues. This suggests that the JA necessary for AO-induced resistance against BCN is also produced in sugar beet shoots.

### AO Application Primes Sugar Beet for Enhanced H_2_O_2_ and PAL Activity Upon BCN Infection

**FIGURE 4:**
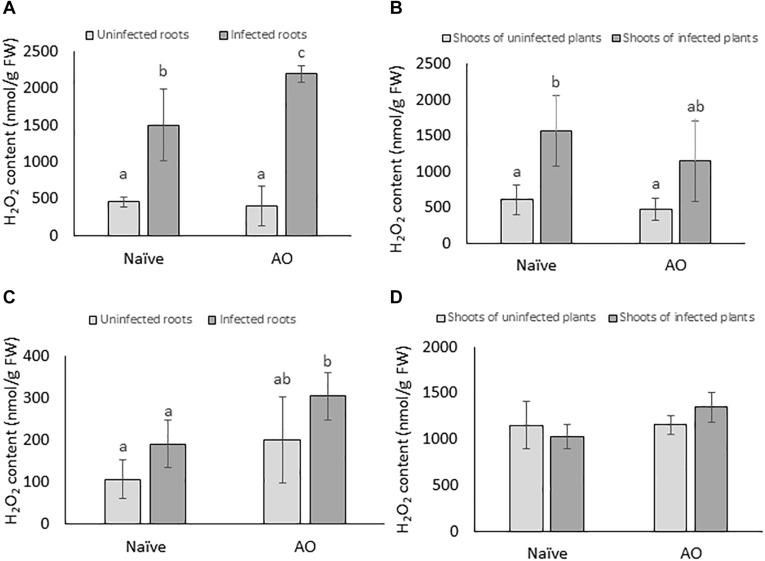
AO induced defense is correlated with primed accumulation of H_2_O_2_ in sugar beet roots. The H_2_O_2_ content per gram of fresh tissue at 4 dpi in **(A)** roots and **(B)** shoots; and at 21 dpi in **(C)** roots and **(D)** shoots. Three-weeks-old plants were sprayed with AO or mock sprayed and 24 h later, plant roots were inoculated with nematodes or mock inoculated. Samples were collected at 4 and 21 dpi. Bars represent the average and SD of four biological replicates, each containing a pool of 4–5 plants. Different letters represent statistically significant differences, while graphs without letters **(D)** had no significant differences between the treatments (Duncan’s multiple range test with α = 0.05).

ROS such as H_2_O_2_ are known to play various roles in basal plant defense responses (Mittler et al., 2004). In this study, we investigated if AO-induced defense is correlated with primed accumulation of H_2_O_2_ upon BCN infection in sugar beet. H_2_O_2_ was measured at 4 and 24 dpi both in root and shoot tissues. When comparing the naïve, infected plants with naïve, uninfected plants a significant higher H_2_O_2_ content was observed in roots and shoots of infected plants at 4 dpi, but not at 24 dpi, showing that BCN infection causes an oxidative burst at early time points (Figures 4A,B). In AO-sprayed uninfected plants, no significant differences in H_2_O_2_ levels were seen, when compared with naïve uninfected plants. However, AO-sprayed infected plants contained significantly higher amounts of H_2_O_2_ in the roots at both 4 dpi (Figure 4A) and 24 dpi (Figure 4C), when compared to roots of naïve infected plants. This phenomenon was not observed in the shoots (Figure 4D). These results imply that AO-defense activation is correlated with a primed accumulation of H_2_O_2_ at the site of infection at both early (4 dpi) and at later time point (24 dpi).

**FIGURE 5:**
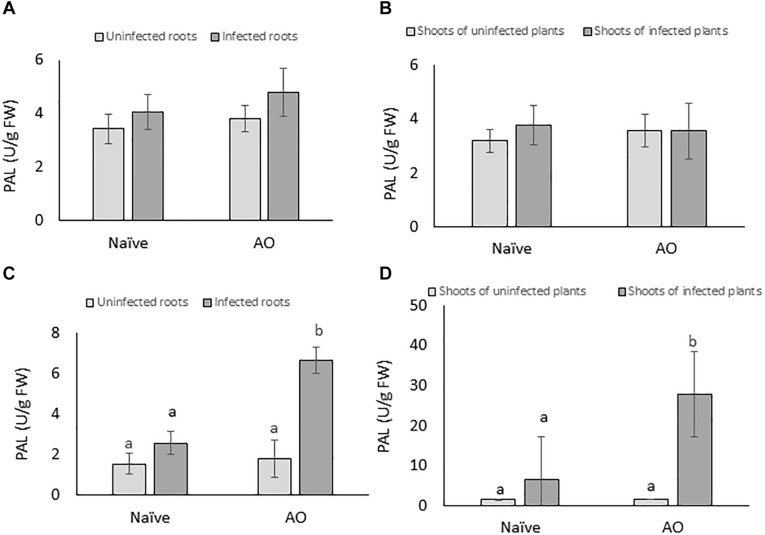
AO induced defense is correlated with phenylpropanoid pathway activation in sugar beet. The PAL enzyme activity per gram of fresh tissue at 4 dpi in **(A)** roots and **(B)** shoots; and at 21 dpi in **(C)** roots and **(D)** shoots. Three-weeks-old plants were sprayed with AO or mock sprayed, and roots were inoculated with nematodes or mock inoculated 24 h after spraying. Samples were collected at 4 and 21 dpi. Bars represent the average and SD of four biological replicates, each containing a pool of 4–5 plants. Different letters represent statistically significant differences, while graphs without letters **(A,B)** had no significant differences between the treatments (Duncan’s multiple range test with α = 0.05).

To investigate if AO-induced defense is correlated with primed activation of PAL, PAL-activity was measured at 4 and 24 dpi for root and shoot tissues of AO-sprayed uninfected and infected plants and compared with naïve uninfected and infected plants. Our results show no significant changes in PAL-activity at the earliest time point (4 dpi) (Figures 5A,B). However, at the later time point (24 dpi), in comparison with naïve plants, we observed a significant increase in PAL-activity in AO-sprayed infected plants, in both roots (∼3× more) (Figure 5C) and shoots (∼4× more) (Figure 5D). This result suggests that PAL activity is primed for activation at later time points upon BCN infection in AO-sprayed sugar beet plants.

## Discussion

Defense priming is known to be induced by different chemical compounds. Primed plants display faster and/or stronger activation of the various defense responses that plants deploy to fight attack by pathogens or pests (Martinez-Medina et al., 2016). For example, the best-known defense priming compound BABA has been shown to induce resistance against PPN in rice, tomato, cucumber, mungbean, and cereals (Oka et al., 1999; Oka and Cohen, 2001; Ahmed et al., 2009; Sahebani et al., 2011; Ji et al., 2015). However, BABA treatment in many plant species results in a stress phenotype that manifests negative effects on growth and development (van Hulten et al., 2006; Koen et al., 2014; Luna et al., 2016) restricting its commercial use. In this research we evaluated the use of another compound that induces plant resistance and defense priming in rice against RKN (Singh et al., 2020)—ascorbate oxidase (AO)—to protect sugar beet plants against BCN *H. schachtii*. Noteworthy, AO does not have negative effects on rice growth. The data reported in this manuscript shows that foliar application of AO results in delayed development of BCN, fewer females and fewer full cysts on the sugar beet host and the treatment even had some positive effects on sugar beet growth. While root-knot nematodes move intercellularly through the roots during their migratory phase, cyst nematodes move intracellularly, creating slightly more damage. On the other hand, while syncytia and giant cells—the feeding sites formed by root-knot nematodes—show functional similarities their ontology is slightly different (Kyndt et al., 2013). Taken together, our results reveal that AO-treatment can protect both dicot sugar beet and monocot rice (Singh et al., 2020) against infection by two groups of nematodes with varying infection strategies without growth penalty effects. Whether this can be extrapolated to other crops and agronomic applications in nematode-infested fields will be an interesting route for further applied research.

It has been shown before that exogenous ascorbic acid (AA) application reduces root infection by the RKN *Meloidogyne incognita* in tomato, and that increased AA levels are present in the roots of *M. incognita* resistant lines (Arrigoni et al., 1979). The authors proposed that AA is utilized for mitochondrial hydroxyproline proteins synthesis to permit development of the cyanide resistant respiration, which is the metabolic process initiated by the cell to potentially counteract the effects of the nematode. Similarly, Fujiwara et al. (2016) showed high accumulation of AA in *Brassica rapa*, a cultivar known to be resistant against *Turnip mosaic virus* (*TMV)*. More recently, we have observed in our own research (Ghaemi et al., in press) that transcripts involved in AA biosynthesis are upregulated in BCN infected roots of resistant plants, but not in susceptible plants, indicating a potential role for AA in sugar beet resistance to *H. schachtii.* In the experiments described in the current manuscript, we showed that foliar application of AO is leading to a primed activation of ROS and PAL enzyme activity in roots infected by BCN. AO regulates the redox status of the apoplastic AA pool (Sanmartin et al., 2002; Pignocchi et al., 2003). It has been postulated that oxidation of apoplastic AA via AO could have the same effect on the apoplastic redox state as an oxidative burst (Foyer and Noctor, 2005). In this work we show that AO-induced defense in sugar beet against BCN, is correlated with a temporal pattern of accumulation of JA, ET and SA in the roots, while the concentrations of ABA were unchanged. IAA was slightly accumulating at 12 h after spraying. This could explain why positive effects on shoot and root growth were observed in AO-treated plants.

Our results with hormone inhibitors showed that AO-induced defense is partially dependent on JA, ET, and PAL activity. Kammerhofer et al. (2015) showed that JA triggers early defense responses in Arabidopsis against *H. schachtii* with significant upregulation of JA/ET marker genes at 24 h after inoculation. Here, we confirmed that JA is also important for defense against *H. schachtii* in sugar beet. Transcriptome analysis and hormone measurement of AO-sprayed rice plants revealed that JA biosynthesis pathway and ET-responsive genes were activated in the rice roots (Singh et al., 2020). Here, we extended and confirmed those observations by revealing JA accumulation at 12 h after AO spraying in sugar beet roots. Interestingly, DIECA application precluded AO-induced defense, confirming an important role for LOX-dependent JA production. Indeed, the gene expression analysis confirmed the aboveground induction of *LOX2* (JA biosynthesis gene) and *RAP2.3* (ET response gene) in AO-treated plants, although these genes were not very strongly induced in root tissues. A significant increase in number of cysts in DIECA treated plants confirms that LOX-mediated JA production plays an important role in sugar beet defense against BCN. However, it is possible that DIECA is not only inhibiting JA production and that *LOX2* is involved in production of other compounds than only JA. For example, Farmer et al. (1994) showed that DIECA inhibits the whole octadecanoid signaling pathway in tomato. JA metabolite cis-(+)-12-oxo-phytodienoic acid (cis-OPDA) was noted as the key signaling molecule in the regulation of Arabidopsis defense against the root-knot nematode *M. hapla* (Gleason et al., 2016). Whether other LOX-derived metabolites are involved in AO-induced defense in sugar beet remains to be elucidated. A full transcriptome and metabolome analysis with multiple time points and tissues, including uninfected and BCN infected sugar beet plants is advised in order to further unravel how AO-induced systemic resistance works. However, the lack of a well-annotated sugar beet genome and detailed functional genetic details of genes and pathways in this understudied crop plant is currently hampering such analyses.

In our experiments we observed that root ET levels are increased at 24 h after AO spraying, the exact moment when we normally execute inoculation. AA is a well-known cofactor for 1-aminocyclopropane-1-carboxylate (ACC) oxidase, the rate-limiting enzyme for ET biosynthesis. Ghaemi et al. (in press) showed that sugar beet plants sprayed with MeJA or with the ET-generator Ethephon (ETH) were significantly less susceptible to BCN when compared to control plants. While our results with the ET-inhibitor confirm a positive role of ET in defense against BCN in sugar beet, ET has been suggested to be critical for syncytium formation during cyst-nematode infection in Arabidopsis (Goverse et al., 2000) and ET levels or signaling were observed to play an important positive role in attraction of *Heterodera glycines* to soybean roots (Hu et al., 2017) and in *H. glycines* development (Tucker et al., 2009). Moreover, the role of ET in parasitism was evidenced by the significant reduction of cyst nematodes inside the roots of ET-insensitive Arabidopsis mutants or wild type plants treated with ET inhibitors (Goverse et al., 2000), an observation which is not confirmed by the results provided in the current manuscript. However, Kammerhofer et al. (2015) demonstrated that BCN are able to interfere with hormone-based defense and signal transduction. ET seems to play different roles at different stages of nematode infection (also reviewed in Kyndt et al., 2013). Indeed, Kammerhofer et al. (2015) showed that ET is only having a role in plant defense at early stages of parasitism, but not in BCN development. In future the levels of ET at other time points after AO treatment, and upon subsequent infection could also be investigated to provide a better time-dependent view on the role of ET in AO-treated plants and in the context of BCN infection.

We also observed an increased concentration of salicylic acid (SA) in AO-sprayed plants at 24 h. Kammerhofer et al. (2015) showed that BCN establishment requires local suppression of the SA pathway. Whereas SA does not play a major role during early *H. schachtii* attraction and infection, it rather acts as a negative regulator during later phases of parasitism. Wubben et al. (2001) also confirmed a role for SA, mediated by NPR1 and negatively regulated by SNI1, in limiting cyst nematode parasitism during a compatible interaction. By applying the Pal-Inh, which specifically blocks the phenylpropanoid pathway responsible for one branch of SA biosynthesis, we were expecting an increase in plant susceptibility for BCN. However, we did not observe significant changes in the number of cysts when compared with control. Two different biosynthetic pathways of SA exist, with one engaging isochorismate synthases (ICSs) and the other PAL, and hence the other pathway could still produce sufficient SA levels. Importantly, the PAL inhibitor AOPP is also known to inhibit auxin biosynthesis (Soeno et al., 2010) and this could have also influenced nematode infection levels. Auxin is required for cyst nematode feeding site development (Grunewald et al., 2009), and thus inhibiting auxin could perhaps lower the number of cysts, complicating the interpretation of this result.

However, when AO is combined with the PAL-Inh, AO is only partially able to protect the plants compared to control plants, indicating that AO-induced defense is partially dependent on PAL. Indeed, we observed a primed increase in PAL-activity in shoots and roots of AO- sprayed plants upon nematode infection, although only at a later time point. Similarly, our previous research (Singh et al., 2019) showed the importance of the phenylpropanoid pathway in activation of systemic defense against RKN in COS-OGA sprayed rice plants. Which specific phenylpropanoid-derived molecule is important for this systemic signaling remains to be elucidated.

One of the earliest defense responses in plants is ROS production in the apoplast (Coll et al., 2011). Our results show a primed accumulation of H_2_O_2_ in roots of AO-sprayed plants at both 4 and 24 dpi with BCN. Similarly, Melillo et al. (2006) and Huang et al. (2016) showed an increased H_2_O_2_ during *M. graminicola* infection in rice and *M. incognita* infection in tomato respectively. In contrast, Siddique et al. (2014) proposed that H_2_O_2_ is needed for successful syncytium formation in Arabidopsis. Increases in H_2_O_2_ were observed in Arabidopsis shoots upon BCN infection at 3 dpi (Siddique et al., 2014). Labudda et al. (2018) showed that the development of *H. schachtii*-induced syncytia in roots alters ROS homeostasis in the shoots of infected plants. Similarly, an increased H_2_O_2_ level was observed in the shoots of nematode-infected sugar beet plants at 4 dpi. The fact that this was not observed in the shoots of these plants at 4 dpi, suggests that in AO-primed plants ROS production is mostly confined to the site of infection (i.e., roots). This local oxidative burst in plant roots is correlated with strongly reduced nematode development in AO-treated plants. ROS act as secondary messengers in the systemic long distance signaling network (Shetty et al., 2008) and in root-shoot communication upon nematode infection (Wang et al., 2019). Communication between aboveground and belowground plant tissues in plant defense against parasitic RKN in tomato was shown to be dependent on an integration between oxidative burst (ROS production), electrical signals and JA synthesis (Wang et al., 2019). The observations reported here indicate that aboveground AO application on shoots is activating DHA accumulation in roots and reveal that this pathway can also protect plants from cyst nematode infection. Application of DIECA, an inhibitor of LOX-dependent JA production, could have inhibited this systemic signaling pathway and hence precluded AO priming activity against BCN. The observation that LOX2 is mainly activated in aboveground tissues, correlates well with the study of Wang et al. (2019) who demonstrated that the JA needed for tomato defense against root-knot nematodes is produced in aboveground tissues. This observation suggests that the JA required for sugar beet defense against BCN is predominantly produced in shoots.

Collectively it can be concluded that AO-induced systemic resistance against BCN is dependent on a time-dependent dynamic pattern of activation of plant defense hormone pathways, with a major role for LOX-dependent JA production. This study shows that AO-induced resistance in sugar beet leads to a primed activation of PAL and H_2_O_2_ levels upon BCN infection. This treatment protects the plant from cyst nematode infection and could potentially provide an extra tool for farmers to control this parasitic nematode in the field.

## Data Availability Statement

All datasets generated for this study are included in the article/Supplementary Material.

## Author Contributions

TK and RS planned and designed the research and wrote the manuscript. RS and NN conducted infection experiments and biochemical analyses. KD and RS did hormone measurements. All authors contributed to the article and approved the submitted version.

## Conflict of Interest

The authors declare that the research was conducted in the absence of any commercial or financial relationships that could be construed as a potential conflict of interest.
