# Supplementary material for: Ascorbate Oxidase Induces Systemic Resistance in Sugar Beet Against Cyst Nematode Heterodera schachtii
Source: Front Plant Sci. 2020 Oct 22;11:591715. doi: 10.3389/fpls.2020.591715 (PMC7641898; doi:10.3389/fpls.2020.591715)
Supplement: Supplementary file 1 [file Presentation_1.PPTX]

## Slide 1
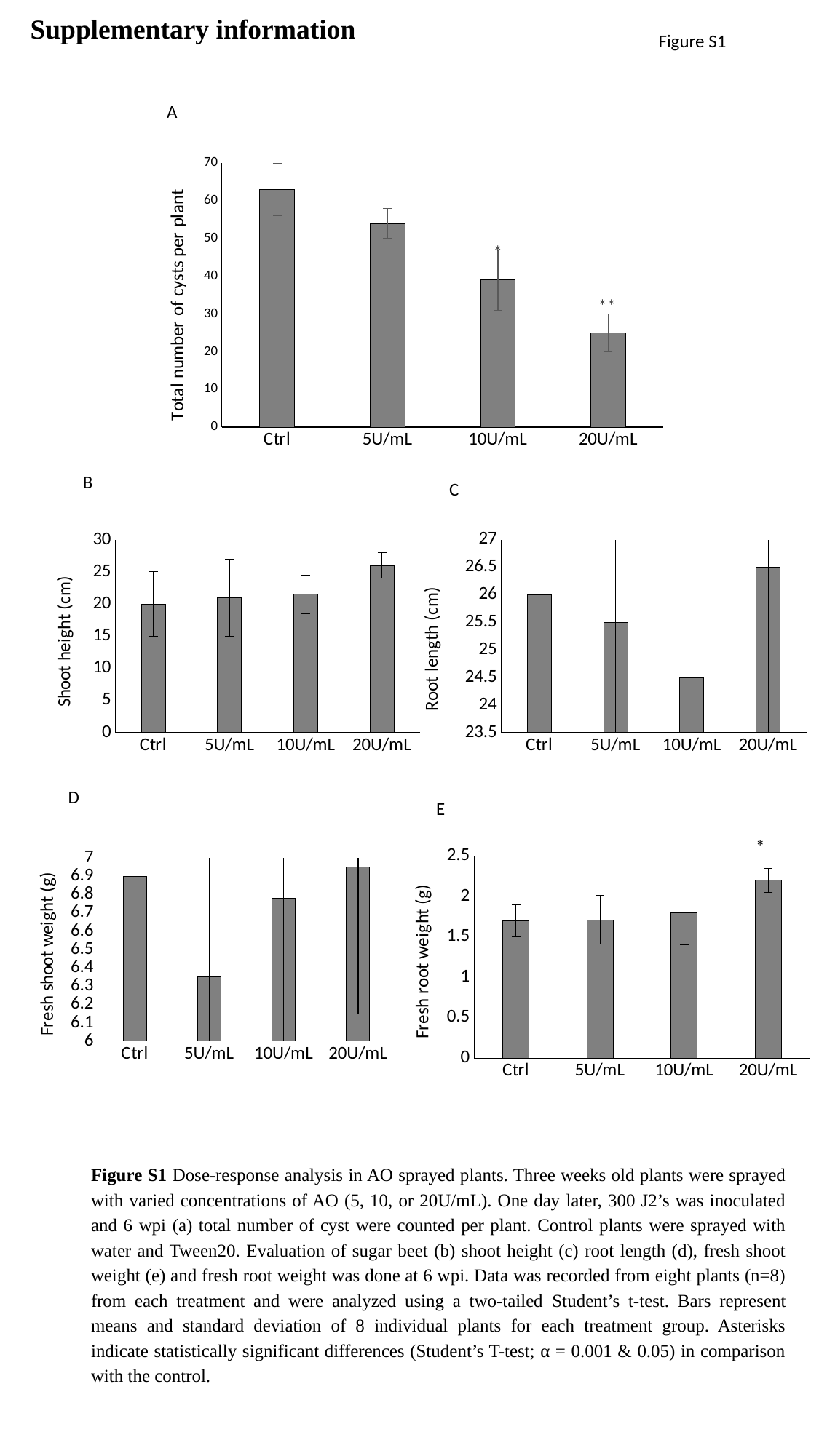

Supplementary information
Figure S1
A
### Chart
| Category | |
|---|---|
| Ctrl | 63.0 |
| 5U/mL | 54.0 |
| 10U/mL | 39.0 |
| 20U/mL | 25.0 |B
C
### Chart
| Category | SH |
|---|---|
| Ctrl | 20.0 |
| 5U/mL | 21.0 |
| 10U/mL | 21.5 |
| 20U/mL | 26.0 |
### Chart
| Category | RL |
|---|---|
| Ctrl | 26.0 |
| 5U/mL | 25.5 |
| 10U/mL | 24.5 |
| 20U/mL | 26.5 |D
E
### Chart
| Category | FSW |
|---|---|
| Ctrl | 6.9 |
| 5U/mL | 6.35 |
| 10U/mL | 6.78 |
| 20U/mL | 6.95 |*
### Chart
| Category | FRW |
|---|---|
| Ctrl | 1.7 |
| 5U/mL | 1.71 |
| 10U/mL | 1.8 |
| 20U/mL | 2.2 |Figure S1 Dose-response analysis in AO sprayed plants. Three weeks old plants were sprayed with varied concentrations of AO (5, 10, or 20U/mL). One day later, 300 J2’s was inoculated and 6 wpi (a) total number of cyst were counted per plant. Control plants were sprayed with water and Tween20. Evaluation of sugar beet (b) shoot height (c) root length (d), fresh shoot weight (e) and fresh root weight was done at 6 wpi. Data was recorded from eight plants (n=8) from each treatment and were analyzed using a two-tailed Student’s t-test. Bars represent means and standard deviation of 8 individual plants for each treatment group. Asterisks indicate statistically significant differences (Student’s T-test; α = 0.001 & 0.05) in comparison with the control.

## Slide 2
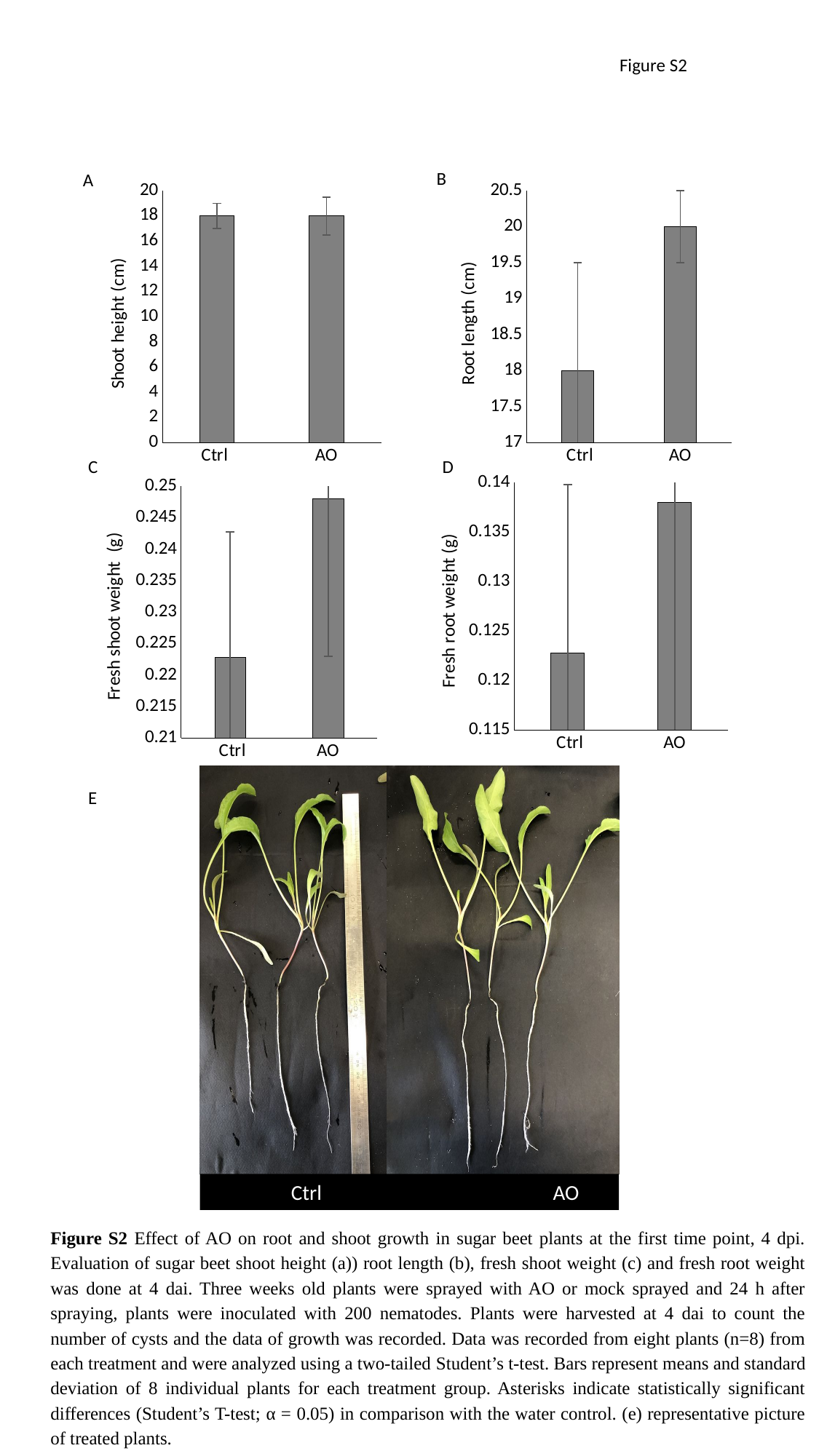

Figure S2
B
A
### Chart
| Category | SH |
|---|---|
| Ctrl | 18.0 |
| AO | 18.0 |
### Chart
| Category | RL |
|---|---|
| Ctrl | 18.0 |
| AO | 20.0 |C
D
### Chart
| Category | Root weight |
|---|---|
| Ctrl | 0.12275 |
| AO | 0.138 |
### Chart
| Category | Shoot weight |
|---|---|
| Ctrl | 0.22275 |
| AO | 0.248 |
 Ctrl
	 AO
E
Figure S2 Effect of AO on root and shoot growth in sugar beet plants at the first time point, 4 dpi. Evaluation of sugar beet shoot height (a)) root length (b), fresh shoot weight (c) and fresh root weight was done at 4 dai. Three weeks old plants were sprayed with AO or mock sprayed and 24 h after spraying, plants were inoculated with 200 nematodes. Plants were harvested at 4 dai to count the number of cysts and the data of growth was recorded. Data was recorded from eight plants (n=8) from each treatment and were analyzed using a two-tailed Student’s t-test. Bars represent means and standard deviation of 8 individual plants for each treatment group. Asterisks indicate statistically significant differences (Student’s T-test; α = 0.05) in comparison with the water control. (e) representative picture of treated plants.

## Slide 3
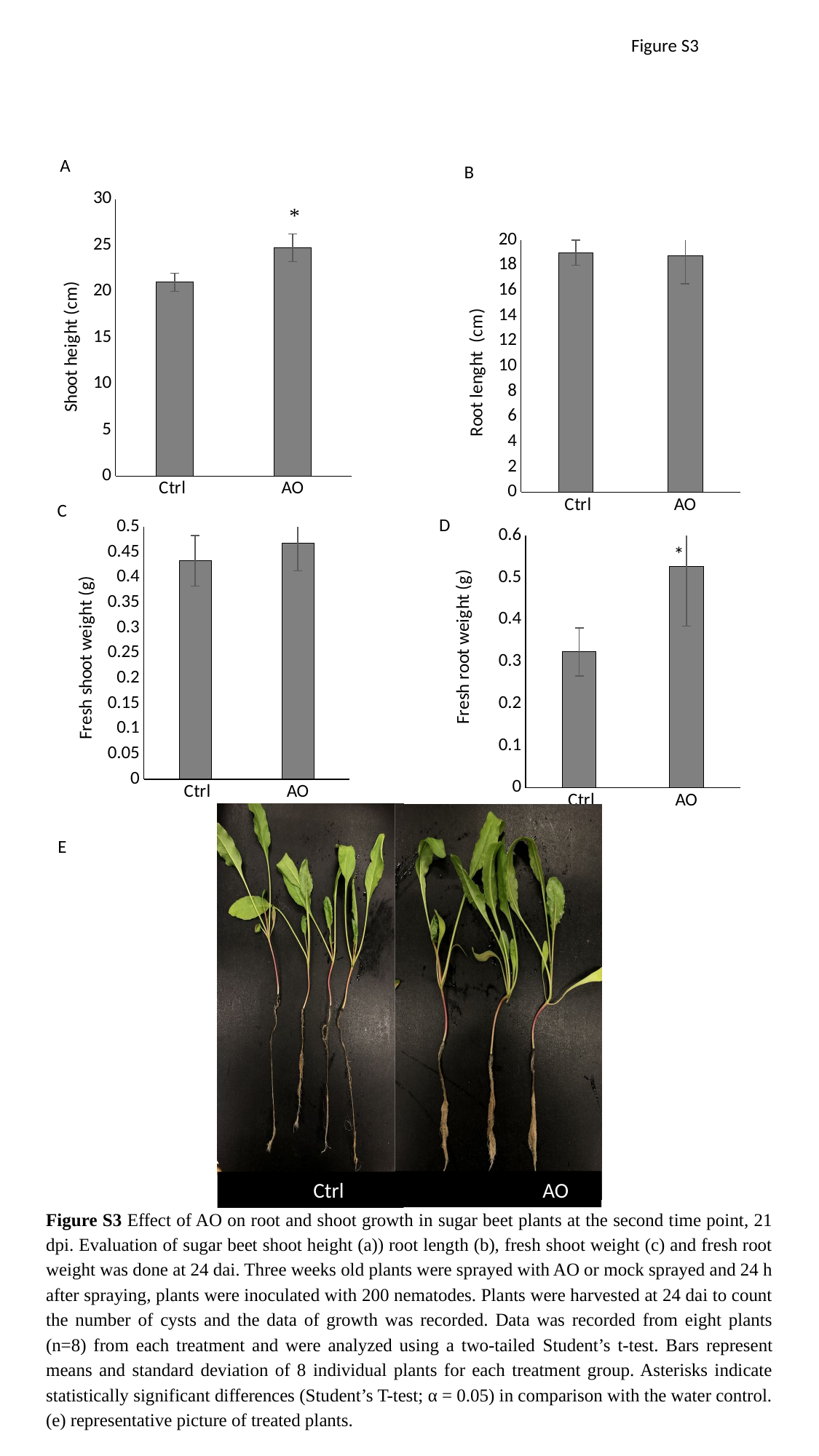

Figure S3
A
B
### Chart
| Category | |
|---|---|
| Ctrl | 21.0 |
| AO | 24.75 |
### Chart
| Category | RL |
|---|---|
| Ctrl | 19.0 |
| AO | 18.75 |C
D
### Chart
| Category | Shoot weight |
|---|---|
| Ctrl | 0.43275 |
| AO | 0.468 |
### Chart
| Category | Root weight |
|---|---|
| Ctrl | 0.3233333333333333 |
| AO | 0.52725 |
 Ctrl
 AO
E
Figure S3 Effect of AO on root and shoot growth in sugar beet plants at the second time point, 21 dpi. Evaluation of sugar beet shoot height (a)) root length (b), fresh shoot weight (c) and fresh root weight was done at 24 dai. Three weeks old plants were sprayed with AO or mock sprayed and 24 h after spraying, plants were inoculated with 200 nematodes. Plants were harvested at 24 dai to count the number of cysts and the data of growth was recorded. Data was recorded from eight plants (n=8) from each treatment and were analyzed using a two-tailed Student’s t-test. Bars represent means and standard deviation of 8 individual plants for each treatment group. Asterisks indicate statistically significant differences (Student’s T-test; α = 0.05) in comparison with the water control. (e) representative picture of treated plants.

## Slide 4
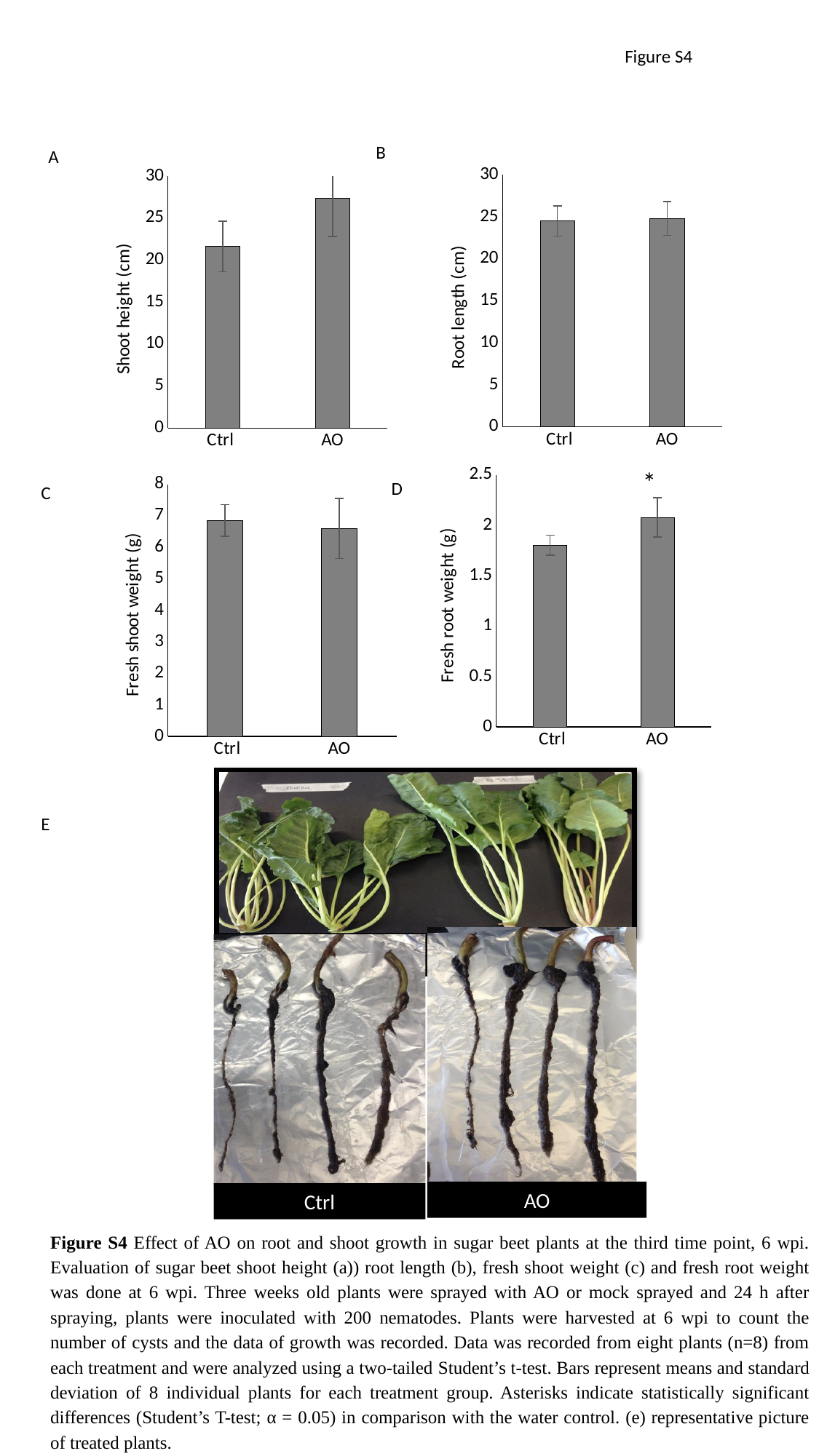

Figure S4
B
A
### Chart
| Category | RL |
|---|---|
| Ctrl | 24.5 |
| AO | 24.8 |
### Chart
| Category | SH |
|---|---|
| Ctrl | 21.6 |
| AO | 27.3 |
### Chart
| Category | Root weight |
|---|---|
| Ctrl | 1.80275 |
| AO | 2.078 |*
### Chart
| Category | |
|---|---|
| Ctrl | 6.856 |
| AO | 6.596 |D
C
 AO
 Ctrl
AO
Ctrl
E
Figure S4 Effect of AO on root and shoot growth in sugar beet plants at the third time point, 6 wpi. Evaluation of sugar beet shoot height (a)) root length (b), fresh shoot weight (c) and fresh root weight was done at 6 wpi. Three weeks old plants were sprayed with AO or mock sprayed and 24 h after spraying, plants were inoculated with 200 nematodes. Plants were harvested at 6 wpi to count the number of cysts and the data of growth was recorded. Data was recorded from eight plants (n=8) from each treatment and were analyzed using a two-tailed Student’s t-test. Bars represent means and standard deviation of 8 individual plants for each treatment group. Asterisks indicate statistically significant differences (Student’s T-test; α = 0.05) in comparison with the water control. (e) representative picture of treated plants.

## Slide 5
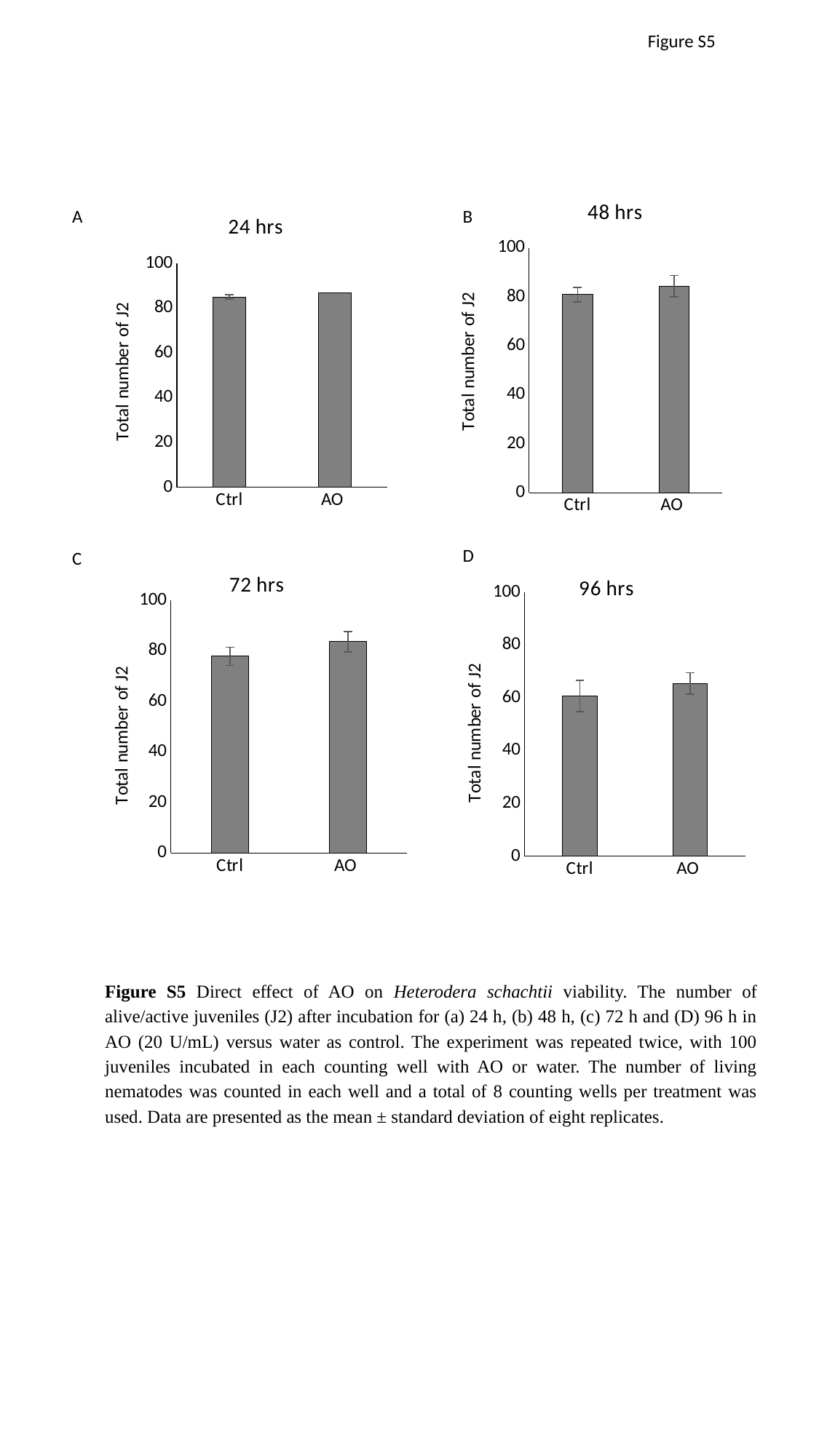

Figure S5
### Chart:
| Category | 24 hrs |
|---|---|
| Ctrl | 85.0 |
| AO | 86.75 |
### Chart:
| Category | 48 hrs |
|---|---|
| Ctrl | 80.875 |
| AO | 84.375 |A
B
D
C
### Chart:
| Category | 72 hrs |
|---|---|
| Ctrl | 77.875 |
| AO | 83.625 |
### Chart: 96 hrs
| Category | 96hrs |
|---|---|
| Ctrl | 60.75 |
| AO | 65.5 |Figure S5 Direct effect of AO on Heterodera schachtii viability. The number of alive/active juveniles (J2) after incubation for (a) 24 h, (b) 48 h, (c) 72 h and (D) 96 h in AO (20 U/mL) versus water as control. The experiment was repeated twice, with 100 juveniles incubated in each counting well with AO or water. The number of living nematodes was counted in each well and a total of 8 counting wells per treatment was used. Data are presented as the mean ± standard deviation of eight replicates.

## Slide 6
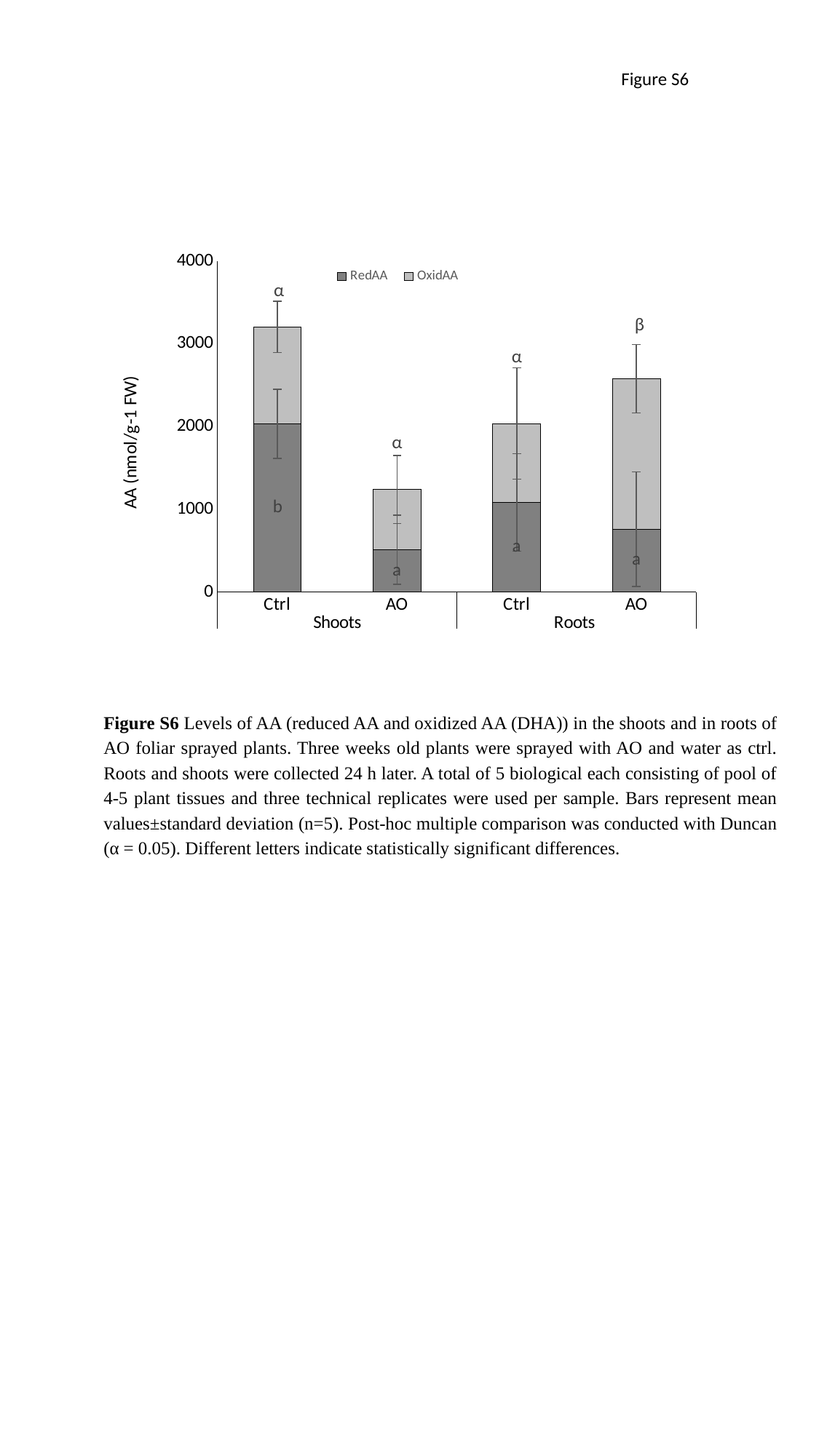

Figure S6
### Chart
| Category | RedAA | OxidAA |
|---|---|---|
| Ctrl | 2034.0 | 1172.0 |
| AO | 512.0 | 729.0 |
| Ctrl | 1084.0 | 954.0 |
| AO | 762.0 | 1817.0 |Figure S6 Levels of AA (reduced AA and oxidized AA (DHA)) in the shoots and in roots of AO foliar sprayed plants. Three weeks old plants were sprayed with AO and water as ctrl. Roots and shoots were collected 24 h later. A total of 5 biological each consisting of pool of 4-5 plant tissues and three technical replicates were used per sample. Bars represent mean values±standard deviation (n=5). Post-hoc multiple comparison was conducted with Duncan (α = 0.05). Different letters indicate statistically significant differences.
